# Use of fosfomycin combination therapy to treat multidrug-resistant urinary tract infection among paediatric surgical patients – a tertiary care centre experience

**DOI:** 10.1099/acmi.0.000163

**Published:** 2020-09-03

**Authors:** Shiju K.S., Gopichand Pallam, Jharna Mandal, Bibekanand Jindal, Kumaravel S.

**Affiliations:** ^1^​ Department of Pediatric Surgery, JIPMER, Puducherry, India; ^2^​ Department of Microbiology, JIPMER, Puducherry, India

**Keywords:** amikacin, combination, complicated urinary tract infection, fosfomycin, meropenem, multidrug-resistant organisms

## Abstract

With increasing resistance to currently used antibiotics, antibiotic combinations are being resorted to. The present study deals with five children with complicated urinary tract infection (UTI) whose urine cultures grew multidrug-resistant (MDR) organisms. In all of these five cases, MDR organisms were the causative agents for UTI and the currently available antibiotics, including colistin, were ineffective, although the organisms were sensitive *in vitro*. In all of these cases, the isolates reverted to being susceptible to the quinolones and cephalosporins tested, namely ceftriaxone and ceftazidime. All were treated using a combination of fosfomycin with other antibiotics, since it has no interference with other classes of antibiotics. Our observations suggest that the use of a combination of fosfomycin with either a carbapenem or an aminoglycoside in a clinical setting would be a reasonable choice to treat UTIs caused by MDR organisms, especially in complicated cases that require chronic therapy.

## Cases

Paediatric surgical conditions such as pelviureteric junction obstruction, posterior urethral valves, vescicoureteral reflex, uretrocele, etc., that produce either structural or functional abnormalities, result in complicated urinary tract infections (UTIs) [1]. Diagnostic invasive procedures such as voiding cystourethrogram and therapeutic procedures such as percutaneous nephrostomy (PCN), cystoscopy may have predisposed the children in this study to develop UTIs [2]. Recurrent UTIs are commonly encountered in such children [3]. Due to their complicated nature, prompt and appropriate treatment is warranted in such cases. With increasing resistance to currently used antibiotics, alternative antibacterials are being resorted to [4–6]. In this context, fosfomycin, which is a broad-spectrum antibiotic with bactericidal activity against multidrug-resistant (MDR) organisms, including extended-spectrum beta-lactamase (ESBL)- and carbapenemase-producing organisms, has been found to be effective [7, 8]. We present five cases involving patients aged 1 month to 12 years whose cultures grew MDR organisms. All were treated using fosfomycin combination therapy.

### Case 1

A 5-year-old child who had recurrent UTI was seen in the outpatient department and had already received amikacin in another hospital before coming to our centre. The child’s urine sample was submitted for culture sensitivity. The culture grew *Enterobacter cloacae *, which was resistant to amikacin, ciprofloxacin, ceftriaxone, ceftazidime, piperacillin/tazobactum, nitrofurantion and meropenem and was only sensitive to colistin [using the microbroth dilution (MBD) method as per the Clinical and Laboratory Standards Institute’s (CLSI’s) 2019 guidelines] [9] and fosfomycin [tested by agar dilution using the *Enterobacteriaceae* breakpoints in the European Committee for Antimicrobial Susceptibility Testing (EUCAST) 2019 guidelines] [10]. Briefly, fosfomycin susceptibility testing was performed using the agar dilution method; Mueller–Hinton agar supplemented with 25 µg ml^−1^ of glucose-6-phosphate was employed and incubated for 18±2 h at 37 °C in ambient air. The minimum inhibitory concentration (MIC) of fosfomycin was defined as the lowest concentration that inhibited the visible growth of the organism. Control strains, including *Escherichia coli* ATCC 25922, and *Pseudomonas aeruginosa* ATCC 27853, were included in each set of tests. Since the patient was ambulatory, colistin was not advised. The child was started on a combination of fosfomycin and ceftriaxone (third-generation injectable cephalosporin). After 3 days of therapy with this combination, a repeat urine culture grew *E. cloacae *, which was sensitive to all the antibiotics. The MICs of ciprofloxacin and ceftriaxone were within the sensitive range. The combination therapy was continued for another 5 days and the final urine culture after 7 days was found to be culture-negative.

### Case 2

A 12-year-old child presented with fever, vomiting and left flank pain. Radiological investigations revealed a 2.5 cm calculus in the left renal pelvis with hydronephrosis. The child was started on cefotaxime and amikacin empirically and as urine became clear, the child underwent retroperitoneoscopy, which was converted to an open nephrolithotomy and stone removal. Prior to starting the therapy, urine culture was not performed. This was followed by uretrocalycostomy with a PCN. The initial culture after the surgery showed the growth of *Klebsiella pneumoniae *, which was resistant to ciprofloxacin, ceftriaxone and ceftazidime. The child received cefoperazone sulbactam in the postoperative period and was discharged with a left PCN catheter *in situ*. After a month the child presented with features of UTI. Two months following surgery the child again presented with features of UTI and was started on cefotaxime and amikacin empirically. The PCN catheter was changed since it was blocked. The urine culture grew *Enterococcus faecalis* that was sensitive to vancomycin and the antibiotic was changed accordingly. The patient was symptomatically relieved and discharged on a prophylactic antibiotic. Two weeks later the child developed UTI again with decreased PCN output. The urine culture this time grew *P. aeruginosa* that was resistant to meropenem but sensitive to colistin and polymyxin B. Colistin injections were administered for 25 days and then a repeat left ureterocalycostomy was performed. Postoperative urine culture again grew *P. aeruginosa* that was only sensitive to colistin (using the MBD method as per the CLSI’s 2019 guidelines) [9] and fosfomycin (tested by agar dilution method as explained in case 1; the *Enterobacteriaceae* breakpoints in the EUCAST 2019 guidelines were used) [10]. Since with colistin no clinical or bacteriological improvement was noted, a combination of fosfomycin and meropenem (carbapenem) was administered. Following the initial 5 days of therapy, another sample was submitted for culture, which grew *P. aeruginosa* with a considerable reduction in colony counts [1000 colony-forming units (c.f.u.) ml^−1^; the initial figure was 100 000 c.f.u. ml^−1^). The child showed remarkable improvement and was finally discharged after a month.

### Case 3

A 1-day-old child presented with bilateral hydronephrosis from birth, having been referred for abdominal distension and respiratory distress. On ultrasound examination the baby had bilateral gross hydronephrosis, affecting the left more than the right. Bilateral ultrasound-guided PCN insertion was performed and the child improved clinically; cefotaxime was then started empirically. A week after insertion, PCN urine culture yielded *E. faecalis* that was resistant to ampicillin and ciprofloxacin, but sensitive to linezolid, vancomycin and nitrofurantoin. The child was started on linezolid based on this report. Ten days following this a repeat PCN sample grew *P.s aeruginosa* that was resistant to all except fosfomycin, and so, based on the experience with case 2, fosfomycin and meropenem (carbapenem) combination was started. The child recovered symptomatically and was discharged after a 1-week hospital stay.

### Case 4

A 7-month-old child with rectovestibular fistula lumbar meningomyelocele, left hydroureteronephrosis and left ureterocele presented with recurrent UTI. The child had previously undergone transverse colostomy for rectovestibular fistula in neonatal period and cystoscopy followed by incision for left ureterocele at 4 months of age. The urine culture grew *K. pneumoniae* that was resistant to third-generation cephalosporins, fluoroquinolones, aminoglycoscides and meropenem. The child was started on piperacillin/tazobactam on an empirical basis. A subsequent urine culture grew *K. pneumoniae* again, but it was sensitive to nitrofurantoin and fosfomycin (tested by the agar dilution method as explained in case 1; the *Enterobacteriaceae* breakpoints in the EUCAST 2019 guidelines were used) [10]. Finally, the child was treated with fosfomycin and ceftriaxone (a third-generation injectable cephalosporin) on which clinical improvement and bacteriological cure were noted.

### Case 5

An 11-month-old child who had been on follow-up for left hydronephrosis detected in the antenatal period presented with features of UTI. The child had an episode of UTI at 5 months of age and on evaluation then had left impaired functioning kidney with vescico-ureteric reflux on the opposite side. The child was started on cefotaxime and amikacin empirically. Initial urine culture grew *E. coli* that was resistant to amikacin, ciprofloxacin, gentamicin, meropenem and ceftazidime and *E. faecalis* that was sensitive to ampicillin, nitrofurantoin and vancomycin and resistant to ciprofloxacin. Based on this, the child was given 5 days of ampicillin parenterally followed by oral suspension of ampicillin for 7 days. But the urine remained turbid and repeat urine culture grew *E. coli* that was sensitive to fosfomycin (tested by the agar dilution method as explained in case 1; the *Enterobacteriaceae* breakpoints in the EUCAST 2019 guidelines were used) [10] and resistant to the rest of the antibiotics tested. Hence the child was started on fosfomycin along with ampicillin (aminopenicillin). Finally, after 7 days of this combination the culture became negative and the patient was discharged.

## Discussion

The selection of antibiotic(s) to treat MDR organisms remains a challenge for clinicians in complicated cases as they require prolonged therapy, which leads to the emergence of resistance. This is complicated further by the formation of biofilms [11, 12]. In all of the five cases in this paper, MDR organisms were the causative agents for UTI and the currently available antibiotics, including colistin, were ineffective, although it was sensitive *in vitro*. Inadequate therapy coverage can increase morbidity and mortality, especially in these complicated cases. Currently, fosfomycin is indicated for the treatment of uncomplicated UTIs. The parenteral fosfomycin has been used to treat systemic infections caused by MDR organisms, including *P. aeruginosa* [13, 14]. Fosfomycin, when used in combination to treat such cases, is usually associated with good clinical outcome and bacteriological cure. In the first child discussed here, the isolate (* E. cloacae *) had reverted to being susceptible to some of the antibiotics tested (ceftriaxone, ceftazidime, piperacillin/tazobactum, meropenem) on repeat culture after the initiation of treatment with a combination of fosfomycin and ceftriaxone. A similar observation was noted in earlier studies performed by Avery *et al*. [15] and Okazaki *et al*. [16]. We do not know the basis of this observation, but we performed a further set of experiments that demonstrated that fosfomycin on its own can alter the resistance pattern of MDR Gram-negative bacteria (GNB), converting them into susceptible ones, and also has the capability to inhibit biofilm formation by MDR GNB [17, 18]. The restoration of susceptibility was enhanced further when a combination of fosfomycin with carbapenems such as meropenem were used [18]. The exact molecular mechanism of the restoration of susceptibility to antibiotics is not known. In an earlier study performed in our centre, the most effective combination was found to be fosfomycin with carbapenems such as meropenem, followed by aminoglycosides such as amikacin, with the less effective combination being with fluoroquinolones like ciprofloxacin [17]. Similar to the *in vitro* observations of the earlier study [17], we observed that in cases 2 and 3 in this paper, the combination of fosfomycin with a carbapenem such as meropenem was effective in attaining bacteriological cure.

These observations suggest that the use of a combination of fosfomycin with either a carbapenem or an aminopenicillin, or even a third-generation cephalosporin such as ceftriaxone, in a clinical setting would be a reasonable choice when tackling UTIs caused by MDR organisms, especially in complicated cases that require chronic therapy.

The possible hypothesis of fosfomycin restoring susceptibility in these MDR organisms is probably due to its capability to increase the permeability of the β-lactams by inhibiting the peptidoglycan recycling intermediates [16, 19]. Further studies need to be performed to understand this.
